# ECHO-CT: An Interdisciplinary Video-Conference Model for Identifying Post-Discharge Transition-of-Care Events

**DOI:** 10.1093/geroni/igaa057.254

**Published:** 2020-12-16

**Authors:** Mariana Gonzalez, Lauren Junge-maughan, Lewis Lipsitz, Amber Moore

**Affiliations:** 1 University of Pennsylvania, Philadelphia, Pennsylvania, United States; 2 Beth Israel Deaconess Medical Center, Boston, Massachusetts, United States; 3 Hebrew SeniorLife and Harvard Medical School, Boston, Massachusetts, United States; 4 Massachusetts General Hospital, Boston, Massachusetts, United States

## Abstract

Introduction: Discharge from the hospital to a post-acute care setting can be complex and potentially dangerous, with opportunities for errors and lapses in communication between providers. Data collected through the Extension for Community Health Outcomes-Care Transitions (ECHO-CT) model were used to identify and classify transitional care events (TCEs.) Methods: The ECHO-CT model employs multidisciplinary teleconferences between a hospital-based team and providers in post-acute settings; during this conference, concerns arising in the patient’s care transition were identified and recorded. Results: 675 patients were discussed during interdisciplinary videoconferences. A total of 139 TCEs were identified; 52 (37.4%) were classified as medication issues, and 58 (41.7%) involved discharge communication or coordination errors. Conclusions: These identified TCEs highlight areas in which providers can work to reduce issues arising in the course of discharge to post-acute facilities. Standardized processes to identify, record, and report transition of care events are necessary to provide high-quality, safe care for patients as they move across care settings.

